# Gamma probes and their use in tumor detection in colorectal cancer

**DOI:** 10.1186/1477-7800-5-25

**Published:** 2008-11-19

**Authors:** Ismet Sarikaya, Ali Sarikaya, Richard C Reba

**Affiliations:** 1 Division of Nuclear Medicine, Georgetown University Hospital, Washington DC, 20007, USA; 2 Department of Nuclear Medicine, Trakya University Hospital, Edirne, Turkey

## Abstract

The purpose of this article is to summarize the role of gamma probes in intraoperative tumor detection in patients with colorectal cancer (CRC), as well as provide basic information about the physical and practical characteristics of the gamma probes, and the radiopharmaceuticals used in gamma probe tumor detection. In a significant portion of these studies, radiolabeled monoclonal antibodies (Mabs), particularly 125I labeled B72.3 Mab that binds to the TAG-72 antigen, have been used to target tumor. Studies have reported that intraoperative gamma probe radioimmunodetection helps surgeons to localize primary tumor, clearly delineate its resection margins and provide immediate intraoperative staging. Studies also have emphasized the value of intraoperative gamma probe radioimmunodetection in defining the extent of tumor recurrence and finding sub-clinical occult tumors which would assure the surgeons that they have completely removed the tumor burden. However, intraoperative gamma probe radioimmunodetection has not been widely adapted among surgeons because of some constraints associated with this technique. The main difficulty with this technique is the long period of waiting time between Mab injection and surgery. The technique is also laborious and costly. In recent years, Fluorine-18-2-fluoro-2-deoxy-D-glucose (18F-FDG) use in gamma probe tumor detection surgery has renewed interest among surgeons. Preliminary studies during surgery have demonstrated that use of FDG in gamma probe tumor detection during surgery is feasible and useful.

## History of gamma probe development

In 1942, Marinelli and Goldschmidt used a hand-held Geiger-Muller (GM) tube to compare uptake of phosphorus-32 sodium phosphate in various skin disorders [1], and later Low-Beer and co-workers used the same technology pre-operatively to differentiate benign from malignant breast lesions [2]. The first intra-operative use of a probe was in patients with brain tumors [3]. Because of the low sensitivity of GM tubes to gamma rays, scintillation probes were developed. Harris et al. reported the use of a thallium-activated cesium iodide scintillation detector and Iodine-131 (^131^I) in patients with thyroid carcinoma undergoing neck exploration [4]. Semiconductor probes became available in the 1970s. Since then a variety of surgical probes have been developed.

There are various surgical probes which can detect X rays, gamma rays (gamma probe) and beta radiation (beta probe). In this review article we will focus on gamma probes. The important performance parameters of a gamma probe includes overall sensitivity (detection efficiency), energy resolution, and spatial resolution [5-7]. Sensitivity is the detected count rate per unit activity. Energy resolution is the ability of the detector to discriminate between radiation with different energies. Energy discrimination is important in separation of primary photons from the scattered photons. It is also important when gamma probe detection is performed with more than one radionuclide having different energies. Spatial resolution is the ability of the detector to determine accurately the location of a source and separate two sources which are close to each other. We will focus this discussion on two types of gamma probes in this article; scintillation-detector and semiconductor ionization detector probes. A scintillation probe consists of a scintillation crystal, a light guide, a photomultiplier tube and associated electronics. Visible light is produced when emitted radiation is absorbed by a stopping medium (a scintillator crytstal), followed by conversion to an electrical pulse. The most commonly used scintillation crystal is thallium-activated sodium iodide (NaI(Tl). There are also thallium-activated cesium iodide (CsI:Tl), and samarium-activated lutecium ortho-oxysilicate (LSO), and bismuth germanate (Bi_4_Ge_3_O_12 _or commonly known as BGO) crystals. A semiconductor ionization detector consists of a semiconductor crystal, a preamplifier and its associated electronics. In semiconductor ionization detectors, free electrons are produced as radiation ionizes the stopping medium (a semiconductor crystal), and the produced electrons are collected as an electrical pulse. The most commonly used semiconducror crystal is cadmium telluride (CdTe). There are also cadmium zinc telluride (CdZnTe), and mercuric iodide (HgI_2_) crystals. Both scintillation and semocinductor probes have distinct relative advantages and disadvantages. Generally, scintillation detector probes have higher sensitivity, particularly for medium to high energy photons and semiconductor probes have better energy resolution and scatter rejection but lower sensitivity, particularly for medium to high energy photons [5,7-9].

The type of the surgical procedure is important in the selection of the most appropriate probe. While excellent spatial resolution (≤ 1 cm) is desired to precisely locate a small lymph node in sentinel node studies, in tumor detection surgeries a probe with high sensitivity will facilitate searching larger areas efficiently [7]. The nuclear characteristics of the radionuclide to be used in gamma probe surgery, emitted photon energy and half-life, are important in the selection of the appropriate probe. While Technetium-99 m (^99m^Tc) labeled agents are mainly used in sentinel node detection surgeries, a wide variety of radionuclides are available for tumor detection surgeries. Side and back shielding of the probe is important where there are high activity sources, such as the injection site, being close to the target area. Thicker shielding, which increases the weight of the probe, is required when higher-energy radiation emitting radionuclides are used. Collimation of the detector provides better spatial resolution but it decreases sensitivity by reducing the effective receiving area of the detector and increasing the minimal distance between the detector and target area [7]. Surgeons prefer to use thin and lightweight probes. However, low weight limits the degree of shielding and collimation. Detector size in current surgical probes, ranges from approximately 5 mm to 32 mm. Smaller detector probes have higher spatial resolution but lower sensitivity than the large detector probes. The shape of the gamma probe is also important in the type of surgery and location of surgical area. A narrow and angled/bent tip gamma probe is more suitable for many studies, mainly sentinel node studies. Wireless (Bluetooth technology) probes eliminates cumbersome cables that can compromise the surgical field and provides the surgeon with operative field flexibility.

An easily operated control unit with clear visual display and a good quality audio signal facilitiates the surgeon's intraoperative work. Recently, flexible imaging probes or portable mini cameras with several centimeters of detector size have been developed for the detection of gamma rays and beta particles in sentinel node and tumor detection surgeries [10-12]. A detector probe is fragile and can be damaged easily if not properly used. The proper care of the surgical probe to protect it from damage, routine quality control, sterilization and electrical safety issues should be rigorously followed.

**Figure 1:**
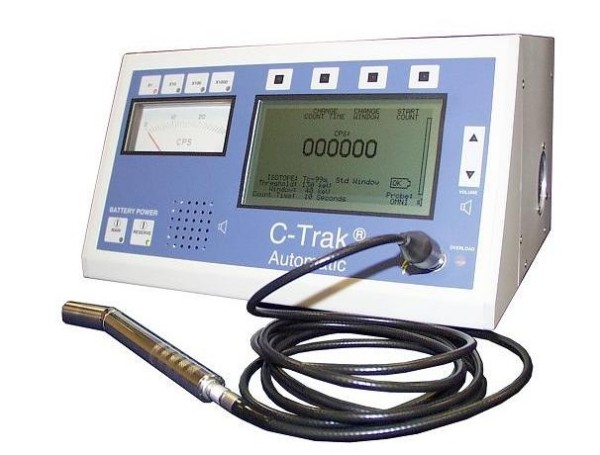
**A basic gamma probe system.** Control unit and probe. (Courtesy of Care Wise Medical Products Corp., CA, US).

A list of commercially available probes in the US with their technical specifications is shown in additional file 1. A basic gamma probe system is shown in figure 1.

## Radiopharmaceuticals used in gamma probe tumor detection surgeries

In a significant portion of the reported gamma probe tumor detection surgeries, radiolabeled Monoclonal Antibodies (Mabs) have been used to localize tumor [13-16]. To a lesser extent, Indium-111 (^111^In) Pentetreotide for neuroendocrine tumors and ^99m^Tc-99m Sestamibi for parathyroid adenomas and breast cancer have been studied [17-22]. In recent years, Fluorine-18-2-fluoro-2-deoxy-D-glucose (^18^F- FDG) has been evaluated for the detection of various cancers via gamma probe [23-26].

### Radiolabeled Monoclonal Antibodies (Mabs)

Various Mabs have been investigated in intraoperative gamma probe radioimmunodetection studies. Among these, anti- tumor-associated glycoprotein-72 (TAG-72) Mabs (B72.3 and CC49) and anti-carcinoembryonic antigen (CEA) Mabs (COL-1, A_5_B_7_, and IMMU-4) have been the most commonly used. TAG-72 is a high-molecular-weight (MW > 10^6^) glycoprotein with characteristics of mucin [27]. It is over-expressed in a wide range of epithelial-derived cancers including colorectal cancer (CRC), breast, gastric, pancreatic, ovarian, and non-small cell lung cancers [27,28]. Increased concentrations have been demonstrated in more than 90% of colorectal, gastric and ovarian carcinomas and approximately 70% of breast cancers [29-32]. TAG-72 is expressed in certain human fetal tissues including fetal intestine but rarely expressed in normal human adult tissues and benign diseases [33]. B72.3, the first-generation murine IgG1 anti-TAG-72 Mab, was shown to be reactive with a wide range of human carcinomas including 94% of CRC, 84% of invasive ductal mammary, 100% of common epithelial ovarian, 96% of lung carcinomas, as well as the majority of gastric, pancreatic, and endometrial carcinomas with only weak or non-reactivity to a wide range of normal adult tissues with the exception of secretory endometrium [29,31-35]. CC49 is a second-generation murine anti-TAG-72 Mab developed at the National Cancer Institute. Studies have demonstrated that CC49 has only minimal immune reactivity to a range of normal tissues but recognized a different epitope on the TAG-72 antigen and exhibited higher reactivity to a wide range of carcinomas including breast, CRC, ovarian, and lung carcinomas compared with B72.3 [35,36]. CC49 demonstrated better tumor detection in patients with CRC [37,38]. Studies indicated that a number of factors, including its pancarcinoma nature, made CC49 a desirable antibody for clinical use [36]. CC83, another second-generation murine Mab against TAG-72, has been shown to have a higher affinity constant than the anti-TAG Mabs CC49 and B72.3. CC83 was found to be safe and sensitive in detecting recurrent CRC [39].

Murine Mabs demonstrate excellent tumor localization but more than 50% of the patients develope a human antimouse antibody (HAMA) response. To prevent this anti-immunoglobulin response, a humanized anti-Tag-72 Mab (HuCC49) was developed [40]. Biodistribution studies demonstrated equivalent tumor-targeting of HuCC49 and CC49 to human colon carcinoma xenografts [40]. This Mab did not produce a HAMA response in any of the studied patients [41].

Another potentially good target antigen in intraoperative gamma probe radioimmunodetection is CEA. It is a cell surface glycoprotein that is over-expressed in a variety of human tumors including CRC, gastric, pancreatic, ovarian, breast and non-small cell lung cancers. Sixty-six to 100% of colon cancers express CEA [29]. Numerous Mabs have been developed by a number of groups to target CEA. COL-1 Mab was shown to have a high K_a _to CEA and to localize to a large number of colon, breast and non-small cell lung cancers [42]. Anti-CEA Mab, A_5_B_7_, a murine IgG1, localized in 97.8% of primary and 88.8% of the principal tumors in second-look procedures in patients with CRC [43]. Another anti-CEA antibody, IMMU-4, a murine IgG1, has been used as well [44,45].

### Technical issues with Mabs

The desirable characteristics of a Mab include; high affinity and avidity for its antigen, easy and rapid penetration to the tumor tissue, long tumor residence time, rapid clearance from the circulation, not accumulating in normal tissues and not producing a HAMA reaction. The form of the antibody, whether whole or fragment, will affect its detection efficiency. Fragments have smaller molecular weight and faster clearance rate from the blood and faster and more efficient penetration to the tumors than the whole antibodies. There is also reduced non-specific antibody binding due to loss of the Fc fragment. All these features result in low normal tissue background activity and increased tumor to background ratio and better detection of tumors. The biodistribution and metabolism of the antibody fragments is also different compared to whole antibody. Whole antibody is more likely to be localized and metabolized in the liver, which compromises efficient detection of liver tumors. Likewise fragments accumulate more in the kidneys and may not be useful in the evaluation of the tumors in or around kidneys and bladder.

Mabs are not truely specific for malignant tissue and can bind to normal tissues and tissues with benign diseases. Antigenic heterogeneity may limit effective detection of cancer using monoclonal antibodies. The use of a monoclonal antibody mixture (antibody cocktail) can enhance targeting of tumor sites. There are controvesial results about possible interfering effect of circulating shed antigens on localization of Mabs in tumor tissue. While some studies report increase in imaging sensitivity when increasing the amount of antibody, some authors did not find a profound difference [46-55]. In a review of the literature, Pimm et al reported that circulating antigen can restrict tumor targeting in human xenografts tumors in nude mice, but this effect is not seen in patients [53]. Shed antigens in the lymphatics may cause misdiagnosis of lymph node metastasis. Most of the Mabs currently used in clinical studies are of murine origin and when given to humans cause HAMA. This may cause rapid clearance of antibody from the circulation before effectively targeting the tumor and limits the possibility of repeat injections. This is more likely with whole antibody injection and less with fragments. It is generally less important in diagnostic studies compared to radio-immunotherapy, where higher amounts of Mabs are used. Genetic engineering techniques have been useful in minimizing the possibility of HAMA by producing recombinant antibodies including chimeric, humanized or human antibodies.

Studies have also investigated biotinylayed Mabs and the avidin system as a method to shorten the current long interval (up to 5 weeks) needed between injection and surgery [56]. There are many pretargeting protocols. The most investigated one involves the avidin-biotin system. Pretargeting is based on the separate administration of cold Mab and the radiolabeled Mab. In order to reduce non-specific uptake of radiolabeled antibody, Mab is first labeled with biotin. The Mab binds to the tumor and non-specific uptake is cleared by RES. Radiolabeled avidin is then injected which localizes in the tumor because of high affinity and specificity of avidin for biotin. A further method of pre-targeting is the hapten/antibody (bispecific antibody) system [57].

### ^18^F-FDG

^18^F-FDG is a positron-emitting non-physiologic analog of glucose. Malignant tumors avidly accumulate FDG because of the accelerated glucose metabolism and increased rate of glucose transport and utilization in malignant cells relative to normal cells. FDG in the blood is transported into the cells via glucose transporters and phosphorylated to FDG-6-phosphate by hexokinase. This is thought to occur more readily in tumors due to overexpression of the glucose transporters GLUT1 and GLUT3 and higher levels of hexokinase in malignant cells [58]. Because glucose-6-phosphatase activity is low in most tissues and in tumors, FDG-6-phosphate cannot be dephosphorylated to FDG and therefore FDG is trapped in the cell. More importantly, FDG-6-phosphate cannot be utilized in the Embden-Meyerhoff cycle of glycolysis resulting in accumulation of the radioactive tracer. In recent years, several groups have utilized ^18^F-FDG for intraoperative gamma probe tumor detection for the detection of tumors in patients with CRC, lung cancer, melanoma, breast cancer, and head and neck cancers including thyroid [23-26]. Approximately 5 to 10 mCi (185 to 370 MBq) of ^18^F-FDG is injected intravenously approximately 30 minutes before the gamma probe surgery. In some cases gamma probe surgery is performed immediately after PET imaging which is approximately 3 hours after injection of 15 mCi (555 MBq) of ^18^F FDG.

### ^99m^Tc-99m Sestamibi

^99m^Tc-99m Sestamibi is used principally for myocardial perfusion imaging. It has been shown to accumulate significantly in various tumors including breast, lung, parathyroid, thyroid, brain and bone. ^99m^Tc-99m Sestamibi is actively transported into mitochondria by the electron gradient between plasma and the mitochondrial membrane potential. This radiopharmaceutical has been used for the detection of parathyroid adenomas by imaging and for intra-operative gamma probe detection. ^99m^Tc-99m Sestamibi is also useful to image P-glycoprotein (Pgp) expression in the tumor cells. Tumors over expressing Pgp pump certain chemotherapeutic agents out of the malignant cells, producing chemoresistance.

Gamma probe surgery can be performed either on the same day following pre-operative imaging (approximately 2.5 to 3 hours following intravenous injection of 15 to 25 mCi (555 to 925 MBq) ^99m^Tc-99m Sestamibi) or in a separate day (approximately 10 min following intravenous injection of 1 mCi (37 MBq) ^99m^Tc-99m Sestamibi) [59].

### ^111^In-111 Pentetreotide

^111^In-111 Pentetreotide (^111^In-111 DTPA-D-Phe) is a peptide that binds to somatostatin receptors, predominantly somatostatin receptor subtypes sst2 and sst5. This peptide localizes in neuroendocrine and some non-neuroendocrine tumors which contain over-expressed somatostatin receptors. These tumors include carcinoid tumors, islet cell tumors, pheochromocytoma, neuroblastoma, paraganglionomas, medullary thyroid cancer, meningiomas, gliomas, and lung carcinoma. Gamma probe surgery is generally performed 24 to 48 hours after the intravenous injection of 4 to 6 mCi (148 to 222 MBq) of ^111^In Pentetreotide.

### Issues with radionuclides used in gamma probe tumor detection surgeries

Many radionuclides are available for labeling tumor targeting agents. Iodine-125 (^125^I) is the most commonly used radionuclide for labeling Mabs. ^131^I, ^111^In, ^99m^Tc, ^123^I, and Thallium-201(^201^Tl) are among the other radionuclides which have been used for this purpose and more recently positron emitters have gained interest in gamma probe tumor detection surgeries.

^125^I is not a suitable agent for imaging due to its low gamma energy, high tissue attention and weak penetration to the tissues, yet its low energy and high soft tissue attenuation is an advantage in gamma probe studies. Low radioactivity concentration in distant organs will improve tumor detection efficiency of the gamma probe when used with ^125^I. When whole Mab is used, the long half-life of ^125^I is an advantage because it takes approximately 14 to 21 days to achieve significant tumor localization.

The beta particle emission of ^131^I contributes significantly to patient radiation absorbed dose. The high energy photons increases background counts and complicates the tumor detection efficiency of the gamma probe. Dehalogenation of the Mab results in free iodide which increases renal activity and compromises its utility for the detection of tumors in or around the kidneys and urinary bladder [60].

The chemistry of ^111^In is similar to that of iron. Following intravenous administration, approximately 30% of free ^111^In in blood is bound to plasma proteins, mainly transferrin. The binding of metals to antibody requires a chelation step to achieve a stable bond. The normal biodistribution patterns of ^111^In labeled agents includes localization in the fixed reticulo-endothelial cells in the liver, spleen, and bone marrow. This can create difficulty when the surgeons attempts to localize small lesions in or near the liver and spleen. However, the technique has been found to be useful in detecting tumor remote from these sites.

## Intraoperative gamma probe tumor detection in CRC

Intraoperative gamma probe tumor detection employs the use of a preoperative injection of a radiolabeled tumor targeting agent and intraoperative detection of tumor via a hand-held gamma probe.

Early experimental work with CEA-producing colon tumor xenografts in swiss nude mice demonstrated that the gamma probe is more sensitive than scintillation camera imaging in detecting small tumors and in detecting tumors with radioactivity levels too low to be imaged [13]. These investigators also reported the preliminary clinical use of a gamma probe in a patient with CRC. In 28 patients with CRC, intraoperative gamma probe radioimmunodetection and ^131^I labeled anti-CEA antibody injection improved sensitivity and specificity over whole body imaging [61]. Subsequent intraoperative gamma probe radioimmunodetection studies, primarily with ^125^I labeled B72.3 Mab, reported that that this method helps surgeons to localize tumors successfully, precisely delinate tumor margins, define the resectability of the tumor, and determine the extent of tumor recurrence [14-16,62,63]. In a group of patients that included colon, gastric, breast and ovarian cancers, intraoperative gamma probe and radiolabeled B72.3 identified tumors in 71% of patients [14]. In a multicenter trial, 104 patients with primary, suspected or known recurrent CRC underwent intraoperative gamma probe radioimmunodetection after injection of ^125^I labeled B73.2 Mab [16]. Intraoperative gamma probe radioimmunodetection localized tumor in 78% of the patients [16]. The overall sensitivity was 77%, and the predictive value of a positive detection was 78%. Intraoperative gamma probe radioimmunodetection and a second generation anti-tumor-associated glycoprotein antibody, ^125^I labeled CC49, successfully localized tumor in 83% of the 36 patients with primary CRC [64]. A pilot study with CC83, another second-generation Mab against TAG-72, provided superior tumor-binding ability and intaoperative localization rates. It was found to be safe with a sensitivity and positive predictive value (PPV) of 100% and 69% respectively as compared to traditional methods that produced 85% sensitivity and 72% PPV [65].

Other studies have demonstrated the role of intraoperative gamma probe radioimmunodetection in detecting occult liver and nodal metastases [16,62,63]. Eighty-six patients underwent exploratory laparatomy with both traditional surgical exploration and intraoperative gamma probe radioimmunodetection following injection of ^125^I labeled CC49 Mab [66]. Arnold et al. in this study reported that intraoperative gamma probe detected more sites of disease compared to traditional surgery in this study. In 41 patients with primary disease traditional exploration detected 45 sites, and intraoperative gamma probe detected 153 sites. In 45 patients with recurrent disease traditional exploration detected 116 sites and intraoperative gamma probe detected 184 sites [66].

A series of publications suggested that decisions based on probe findings changes in patient management result in improved care. Major abdominal surgery can be avoided and patients directly proceed to chemotherapy or radiotherapy and important modifications can be made in the surgical procedure [15,16,62-64]. In one such, intraoperative gamma probe radioimmunodetection findings resulted in staging changes in 34% of the patients, new findings resulted in operative changes in 25% patients, and 30% of the patients became eligible for adjuvant chemotherapy [64].

Other studies have demonstrated that some tissues that were intraoperative gamma probe radioimmunodetection positive were negative on routine hematoxylin and eosin (H&E) staining, but demonstrated micrometastasis when evaluated by more sophisticated immunhistochemistry [67-69]. Identification of lymph nodes with microscopic tumor and/or shed antigen by intraoperative gamma probe radioimmunodetection can be used to reproducibly identify tumor-reactive lymph node lymphocytes for use in adoptive immunotherapy programs and in studying the regulation of immune responses in vivo [70,71].

Several studies have suggested that intraoperative gamma probe radioimmunodetection may improve survival by providing a method of immediate intraoperative staging that might lessen recurrences and lead to early institution of adjuvant therapy [63,72-74]. Eighty-six CRC patients who entered a intraoperative gamma probe radioimmunodetection protocol study with ^125^I labeled B72.3 Mab injection were evaluated for survival following second-look surgical procedures [72]. The median survival was 60+ months for the resectable group, 18 months for the traditional nonresectable group, and 29 months for the intraoperative gamma probe radioimmunodetection nonresectable group. The 2-year survival rates were 95%, 36%, and 57%, respectively, and the 5-year survival rates were 60%, 0%, and 0% [72].

An intraoperative gamma probe radioimmunodetection study with biotinylayed cold Mabs and subsequent injection of radiolabeled avidin localized tumor in 65% of the patients also allowed the surgeon to identify subclinical tumors [75]. This system reduced the mean time interval between antibody injection and surgery to 7 days [75]. In an unrelated study, B72.3 was found to be more sensitive for detection of recurrent cancers and biotinylated anti-CEA Mab FO23C5 to be more effective in patients with primary tumors [76].

In recent years, ^18^F-FDG use in gamma probe tumor detection surgeries has been gaining interest among surgeons. The main advantage of the intraoperative gamma probe over that of preoperative FDG-PET imaging is the ability to have the intraoperative gamma probe in close proximity to the suspected site of recurrent disease at the time of surgery.

Intraoperative gamma probe tumor detection with FDG injection has been shown to correlate well with preoperative FDG-PET findings in patients with CRC [23,77,78]. Intraoperative gamma probe removal of all ^18^F-FDG positive tissue ensures surgeons of more complete removal of the tumor burden as compared to the surgeons traditional approach of assessing and resecting presumed sites of tumor. Essner et al. used ^18^F-FDG and the intraoperative gamma probe in differentiating normal tissue from tumor in patients with metastatic CRC or melanoma [78]. Recently, we demonstrated that combined use of preoperative FDG-PET and intraoperative gamma probe is potentially helpful to the surgeon as a roadmap for accurately locating and determining the extent of tumor recurrence in patients with CRC. While use of the intraoperative gamma probe appeared to be more sensitive in detecting the extent of abdominal and pelvic recurrence, preoperative FDG-PET imaging was more sensitive in detecting liver metastases [26].

In more recent years, Mabs labeled with PET isotopes have been studied. Strong et al. recently reported the use of Iodine-124 (^124^I) labeled humanized Mabs specific for CRC (huA33) and renal tumors [79].

## Conclusion

Intraoperative gamma probe tumor detection of a radiolabeled tumor targeting agent is a promising technology. Intraoperative gamma probe use may enable the surgeon to accomplish a more complete removal of the tumor burden as compared to the surgeons traditional approach of assessing and resecting presumed sites of tumor. Further developments in probe technology and improvement in tumor targeting techniques are on-going to improve sensitivity of tumor detection and management of patients with CRC as well as a number of other malignant tumors.

## Competing interests

The authors declare that they have no competing interests.

## Authors' contributions

IS organized, wrote and revised the manuscript. AS and RCR assisted in the writing and editing of all aspects of this manuscript. All authors read and approved the final manuscript.

## Supplementary Material

Additional file 1 **Commercially available surgical probes in US with specifications.** The data provided show the commercially available surgical probes in US with specifications. Click here for file
