# Supplementary material for: Gamma probes and their use in tumor detection in colorectal cancer
Source: Int Semin Surg Oncol. 2008 Nov 19;5:25. doi: 10.1186/1477-7800-5-25 (PMC2596150; doi:10.1186/1477-7800-5-25)
Supplement: Additional file 1 — Commercially available surgical probes in US with specifications. The data provided show the commercially available surgical probes in US with specifications. [file 1477-7800-5-25-S1.doc]

**Models Detector Material Detector Size Energy**

**Crystal Probe System** **γ probes:**

Nuclear Fields (USA) Corp. Standard straight, flexible, CsI 10-15 mm 50-511 keV

IL, USA laparoscopic

**C-Trak^R^(OmniProbe^R^) γ probes:**

Care Wise Medical Products Corp. Standard angled, laparoscopic CsI(Tl) 11 & 10 mm 27-364 keV

CA, USA PET 25.4 mm 511 keV

**Gammed**   **γ probes :**

Capintec Inc, Small probe (angled) CdTe or CdZnTe 11mm 20-170 keV

NJ, USA Large probe (angled) CsI(Tl) 16 mm 110-1000 keV

Endoscopic probes CdTe

**Gamma Finder^R^** **γ probes :**

World of Medicine, Germany Cordless probe Semiconductor

Silicon Instruments GmbH, Germany

Subsidiary SenoRx Inc. CA, USA

**Navigator^TM^**   **γ probes:**

RMD instruments LLC Standard angled&straight lymphatic, CdTe 10-14 mm up to 364 keV

MA, USA superficial H&N, thoracic,

abdominal

PET 32 mm 511 keV

**Neoprobe**  **γ probes:**

Neoprobe Corp. Straight ( corded & cordless) CdZnTe 14mm 27-364 keV OH, USA Angled ( cordless ) CdZnTe 14 mm 27-364 keV Laparoscopic ( straight & corded) CdZnTe 11 mm 27-600 keV

**Node Seeker^TM^**  **γ probes:**

*IntraMedical Imaging LLC Standard-tip, narrow-tip, bent-tip LYSO 0.25-0.5 inch 20-511 keV

CA, USA high energy (F-18)

**β probe ( positron):** Plastic Scintillator  **Investigational:**

Flexible and mini γ and β cameras

**____________________________________________________________________________________**

γ: Gamma Probe, β: Beta Probe

*GE Healthcare is the exclusive distributor of Node Seeker in US.
